# Chronic Bicompartmental Bucket-Handle Meniscal Tears Associated With Anterior Cruciate Ligament (ACL) Rupture in an Epileptic Patient: A Case Report

**DOI:** 10.7759/cureus.99310

**Published:** 2025-12-15

**Authors:** Basil Alsuwaine, Rahaf Alrasheed, Suha Alanazi, Mohammed Alorayyidh, Ibrahim Assiri

**Affiliations:** 1 Orthopaedic Surgery, King Fahad Medical City, Riyadh, SAU; 2 Orthopaedic Surgery, King Saud University, Riyadh, SAU; 3 Orthopaedic Surgery, Princess Nourah Bint Abdul Rahman University, Riyadh, SAU

**Keywords:** acl tear, arthroscopy, bicompartmental bucket-handle meniscus tear, chronic knee instability, neurological comorbidities

## Abstract

Bilateral bucket-handle meniscal tears (BHMTs) are uncommon, as these injuries are typically unilateral in a single knee and often associated with anterior cruciate ligament (ACL) tears. We present a rare case of a 36-year-old male with a history of intracerebral hemorrhage and epilepsy who developed chronic right knee pain, instability, and mechanical locking. MRI revealed ACL rupture and a displaced meniscal fragment. Arthroscopy confirmed bicompartmental BHMTs and ACL deficiency. Partial meniscectomy and debridement were performed. ACL reconstruction was not undertaken, as arthroscopy showed reattachment of the ligament, adequate graft tightness, and no clinical evidence of instability. ACL reconstruction was not performed due to adequate reattachment of the ACL. This case describes a rare presentation of chronic bicompartmental BHMTs with ACL rupture in a patient with underlying neurological deficits, where the neurological comorbidity likely masked symptoms and contributed to delayed seeking of medical care and delayed diagnosis. This case highlights the diagnostic challenges and therapeutic approach to bicompartmental bucket-handle tears in patients with chronic ACL deficiency and neurological comorbidity.

## Introduction

A bucket-handle meniscal tear (BHMT) is a vertical or oblique longitudinal tear in which a central meniscal fragment remains attached to the anterior and posterior horns but displaces toward the intercondylar notch, away from the peripheral rim [1]. It is highly prevalent in the setting of anterior cruciate ligament (ACL) tears; in 69.8% of cases, reflecting the substantial meniscal burden in this typically young, active population [2]. Meniscal tears are frequent, and the medial meniscus is affected approximately threefold more often than the lateral meniscus. Nevertheless, bicompartmental bucket-handle tears are documented in only about 30% of cases, representing a distinctly rare subtype of meniscal injury [3,4]. The knee menisci play a key biomechanical role in load distribution, shock absorption, joint stability, and lubrication; loss of meniscal tissue, particularly after subtotal or total meniscectomy, has been shown to accelerate degenerative changes and predispose to early osteoarthritis [5]. Clinically, meniscal tears usually present with localized joint-line pain, swelling or effusion, and mechanical symptoms such as catching, clicking, or true locking of the knee, especially during weight-bearing flexion and rotation [6]. The overarching aim of meniscal repair is to relieve symptoms while preserving native meniscal tissue in order to restore near-normal knee biomechanics and protect the articular cartilage [7]. We report a case of bicompartmental BHMT in a 36-year-old man, a presentation that has been rarely reported in the literature.

## Case presentation

A 36-year-old right-leg-dominant man with a history of intracerebral hemorrhage, epilepsy, left-sided lower limb weakness, and bronchial asthma presented to our orthopedic clinic with progressive right knee pain and mechanical symptoms. Two months earlier, he had sustained a twisting injury to the right knee while walking. Since then, he reported new-onset medial knee pain associated with episodes of locking, during which the knee became stuck in flexion and required passive manipulation to extend. He denied acute swelling, redness, fever, constitutional symptoms, or recent seizures, with a previous right knee injury eight years earlier while playing football. At that time, he experienced pain, recurrent locking, and occasional giving-way episodes of the right knee but did not undergo imaging or surgical treatment. Symptoms partially improved after a course of physiotherapy, and he returned to his usual daily activities. For the last two years, he had resumed recreational football without instability until the recent twisting episode. At presentation, he was no longer participating in sports but was able to walk independently without assistive devices, although he remained limited by intermittent locking and pain.

On physical examination, he weighed 62 kg and ambulated with a non-antalgic gait. There was no obvious deformity or effusion of the right knee. Residual neurological deficits from his prior intracerebral hemorrhage were evident, with baseline weakness of the left lower limb. Manual muscle testing showed quadriceps strength of grade 4/5 on the right and 3/5 on the left. The right knee had full extension, with flexion reproducing medial joint-line discomfort, although no mechanical block was noted during the examination. There was localized tenderness along the medial joint line, but no warmth or joint erythema. Ligamentous assessment of the right knee revealed a stable joint with grade 1 anterior drawer and grade 1 Lachman tests, both with a firm endpoint, and no varus or valgus laxity. Distal neurovascular examination was intact.

**Figure 1:**
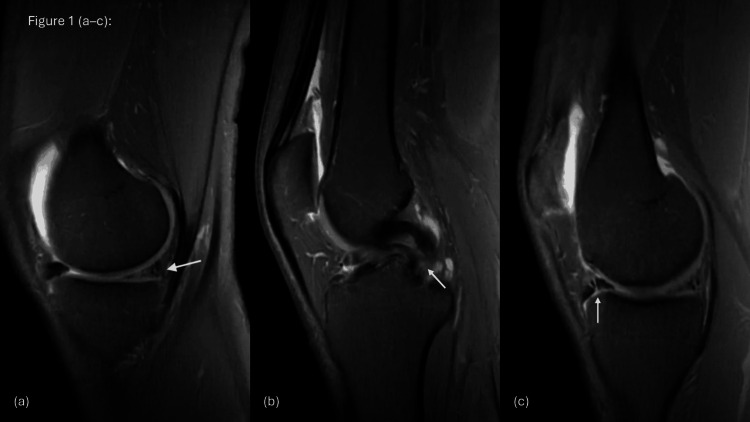
Sagittal T2-weighted MRI showing (a) the ghost sign (white arrow), (b) the double posterior cruciate ligament (PCL) sign (white arrow), and (c) a displaced anterior horn fragment (white arrow).

**Figure 2:**
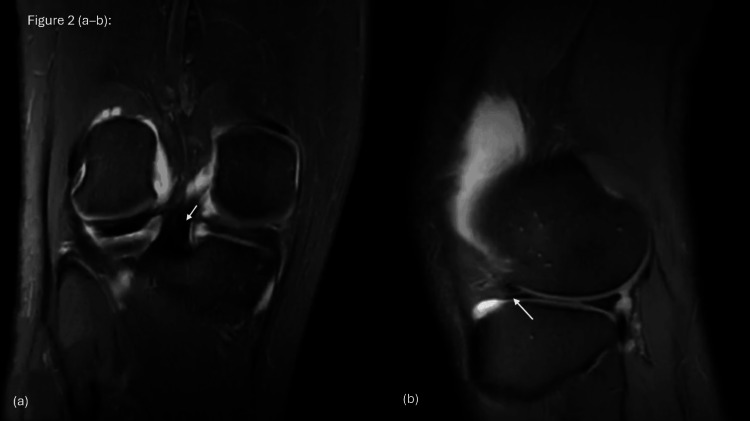
MRI findings showing (a) coronal T2-weighted FSE FS images demonstrating a medial meniscus tear (white arrow), and (b) sagittal T2-weighted FSE FS images showing a lateral meniscus anterior horn tear (white arrow). FSE FS: fast spin-echo fat-suppressed

The pre-operative magnetic resonance imaging (MRI) of the right knee (Figures 1A-1B) demonstrated a large longitudinal tear of the medial meniscus with a flipped fragment extending into the intercondylar notch, consistent with a bucket-handle tear (Figures 1A-1C). The ghost sign and double posterior cruciate ligament (PCL) sign were both observed (Figures 1B-1C). The lateral meniscus appeared intact (Figures 2A-2B). The cruciate ligaments showed a full-thickness ACL tear from the femoral attachment, while the PCL remained intact, with no anterior tibial translation. Minimal periligamentous edema was noted around the medial collateral ligament (MCL), indicating a grade I sprain, whereas the lateral collateral ligament (LCL) was unremarkable. The extensor mechanism, including the quadriceps tendon, patellar tendon, and Hoffa’s fat pad, was normal. Cartilage assessment demonstrated a mildly heterogeneous signal in the medial compartment, with preservation of the lateral and patellofemoral compartments.

The patient underwent subtotal medial and lateral meniscectomy without concomitant ACL reconstruction because the ACL had become fibrotic and reattached to an abnormal location, and the patient had already adapted to this chronic condition.

**Figure 3:**
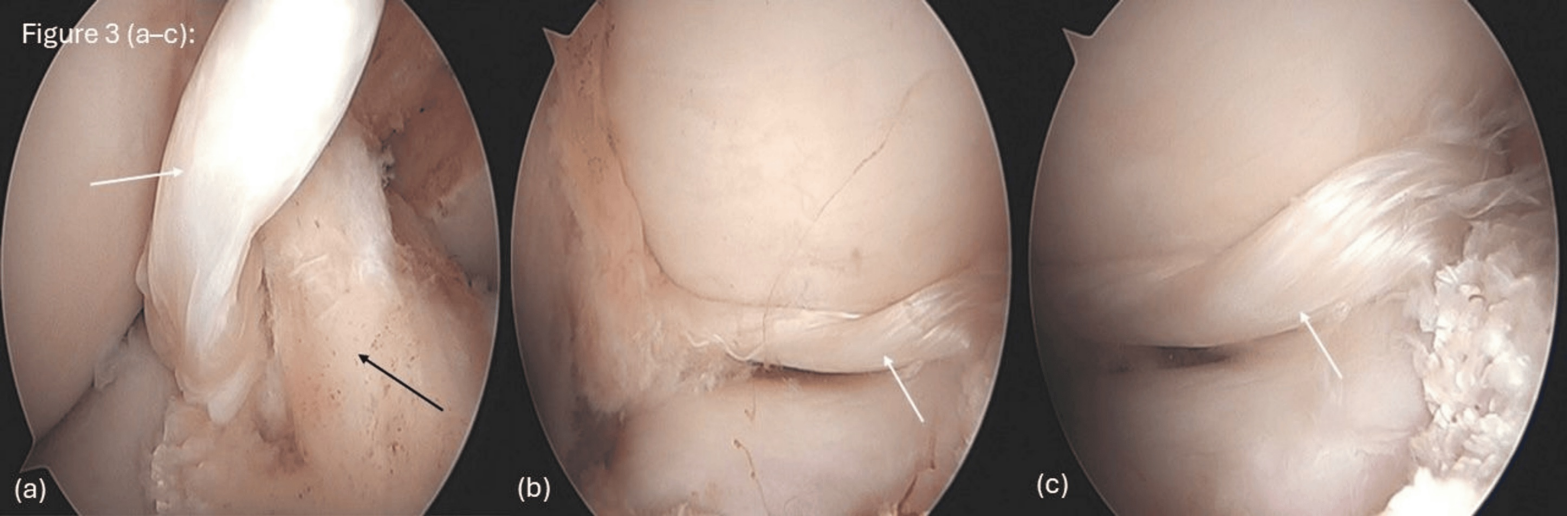
Images showing (a) a lateral meniscus tear (white arrow) with ACL (black arrow), (b) a bucket-handle tear in the lateral compartment (white arrow), and (c) a displaced lateral meniscus fragment in the notch (white arrow). ACL: anterior cruciate ligament

**Figure 4:**
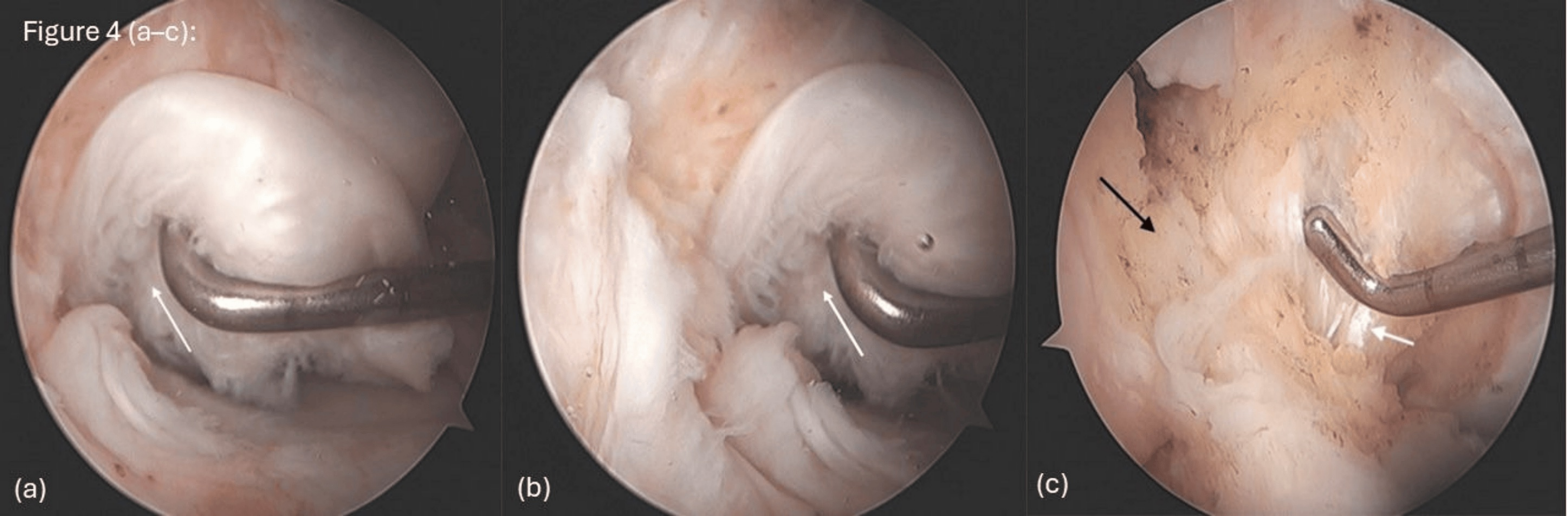
Images showing (a) a medial meniscus tear (white arrow), (b) a bucket-handle tear in the medial compartment (white arrow), and (c) ACL reattachment, with the ACL indicated by the black arrow and the PCL indicated by the white arrow. ACL: anterior cruciate ligament; PCL: posterior cruciate ligament

The bucket-handle tears in both the medial and lateral menisci were confirmed on arthroscopy, with displaced fragments extending into the intercondylar notch (Figures 3A-3C). The degenerative nature of the meniscal tissue precluded repair, necessitating subtotal meniscectomy and joint debridement. Arthroscopic shavers and punches were utilized to remove unstable fragments and contour the remaining meniscal rims. In the lateral compartment, arthroscopy demonstrated a visible meniscus tear (Figure 3A), a bucket-handle configuration (Figure 3B), and a displaced fragment located within the intercondylar notch adjacent to the ACL (Figure 3C). In the medial compartment, a degenerative meniscal tear was observed (Figure 4A), along with a bucket-handle tear (Figure 4B). The ACL was reattached in a more inferior position at the femoral insertion, but intraoperative probing revealed good graft tightness, and thus ACL reconstruction was not performed (Figure 4C).

After surgery, early postoperative assessment showed a right knee range of motion from 0° to 130°. The patient reported overall improvement in his symptoms, although he continued to experience residual pain of about 5/10 on the visual analog scale. He began a supervised rehabilitation program at a private physiotherapy center, which included knee range-of-motion work, quadriceps and hamstring strengthening, neuromuscular training, and adjunctive shockwave therapy. On examination, the anterior drawer and Lachman tests remained grade 1 with a firm endpoint, similar to his preoperative findings, and the knee was clinically stable without any episodes of giving way. At his six-month follow-up, he noted mild but clear improvement in pain, complete resolution of locking, and no further instability during daily activities. He continued to walk independently without the need for assistive devices.

## Discussion

Bilateral BHMTs with an ACL rupture are extremely rare, and this makes the diagnosis difficult, especially when the symptoms do not follow the usual pattern [1]. Our patient was a 36-year-old man who had delayed mechanical symptoms and left-sided neurological deficits. This is different from what has been reported in most cases, where patients typically present soon after injury with obvious locking and instability, as described by Yahyazadeh et al. [8] and Idrissi et al. [9]. In our case, the symptoms appeared almost a year after a secondary twisting event and six years after the original trauma, which contributed to the diagnostic delay.

MRI revealed classic signs of BHMTs, including the ghost sign and the double-PCL sign (Figures 1A-1C). These findings are consistent with what has been reported by Valderrama et al. [2] and Yahyazadeh et al. [8]. Sohail et al. [10] also highlighted the value of these signs, especially in acute injuries. However, similar to other reports, MRI did not fully demonstrate the chronic or complex aspects of the injury. The lateral meniscal tear, for example, was only clearly identified during arthroscopy (Figures 3A-3C), showing that imaging alone can sometimes miss important details.

During surgery, we performed a subtotal medial and lateral meniscectomy with joint debridement because the meniscal tissue was too degenerated to repair. This approach is similar to what was used in other chronic cases where repair was not possible [10,11]. In contrast, younger patients with acute injuries, as described by Bong and Lee [11] and Sohail et al. [10], were suitable for all-inside meniscal repair techniques.

We chose not to reconstruct the ACL because the patient had already adapted to the injury over several years, had neurological deficits, and did not participate in high-demand physical activities. This decision is supported by Wright et al. [12], who reported that partial meniscectomy alone can be enough in non-athletic patients who remain functionally stable despite ACL insufficiency.

The patient recovered well after surgery, began physiotherapy early, and did not experience any complications. His postoperative course is similar to what has been reported in other cases treated either surgically or conservatively [10,11].

Overall, this case shows that MRI has important limitations in chronic meniscal injuries and that arthroscopy remains essential for confirming the diagnosis and guiding management. It also demonstrates how neurological deficits can mask typical knee symptoms and delay diagnosis, and highlights the importance of tailoring treatment, especially decisions regarding ACL reconstruction, to the patient’s functional level, chronic adaptations, and overall condition.

## Conclusions

Chronic bicompartmental BHMTs with ACL insufficiency are extremely uncommon, especially in patients with neurological conditions that may mask typical knee symptoms. In long-standing injuries where the meniscus cannot be repaired, partial meniscectomy can still provide meaningful improvement and acceptable short-term results. In this case, ACL reconstruction was not performed because intraoperative assessment showed that the knee remained relatively stable and the patient had functionally adapted over time. Overall, this case highlights the need for individualized treatment decisions and reinforces the important role of diagnostic arthroscopy in managing complex meniscal injuries.
